# Characterization of the complete plastid genome of *Scutellaria microviolacea* (Lamiaceae), a species endemic to Yunnan Province of China

**DOI:** 10.1080/23802359.2022.2069521

**Published:** 2022-05-06

**Authors:** Yong-Chao Wang, Zhi-Rong Zhang, Li-Jun Yan

**Affiliations:** a School of Vocational and Technical Education, Yunnan Normal University, Kunming, China; b Germplasm Bank of Wild Species, Kunming Institute of Botany, Chinese Academy of Sciences, Kunming, China

**Keywords:** *Scutellaria microviolacea*, endemic species, Lamiaceae, plastid genome, phylogeny

## Abstract

The complete plastid genome of *Scutellaria microviolacea* C. Y. Wu was firstly reported. The full length of the plastid genome was 152,092 bp and comprised of a large single-copy (LSC) region of 84,090 bp, a small single-copy (SSC) region of 17,534 bp, and two inverted repeat regions (IRs) of 25,234 bp. A total of 131 genes were encoded, including 87 protein-coding genes, 36 tRNA genes, and 8 rRNA genes. The overall GC content of the *S. microviolacea* plastid genome was 38.3%. Further phylogenetic analysis based on 18 accessions inferred that the genus *Scutellaria* can be divided into two clades, and *S. microviolacea* is evolutionarily close to *Scutellaria tsinyunensis*. Our study provided essential genetic resources for further studies on the evolution and genetic diversity of the genus *Scutellaria* and its related taxa.

The genus *Scutellaria* belongs to the subfamily of Scutellarioideae (Lamiaceae), which contains about 360 species and is widely distributed in East Asia, Europe, and the United States (Paton 1990; Paton et al. 2016). Many species of *Scutellaria* possess a wide range of pharmacological actions, such as antitumor, antioxidant, anticonvulsant, antibacterial, antiviral, anti-angiogenesis, and hepatoprotective activities (Shang et al. 2010). The infrageneric classifications and species delimitation of this genus remain to be controversial (Shang et al. 2010; Zhao et al. 2021). *S. microviolacea* C. Y. Wu 1977 is an endemic species from Yunnan Province of China (Wu 1977). In the present study, we assembled and characterized the first complete plastid genome of *S. microviolacea* and performed the phylogenetic analysis with other 13 congeneric species.

The sample of *S. microviolacea* was collected from Germplasm Bank of Wild Species, Kunming Institute of Botany, Chinese Academy of Sciences. Total genomic DNA was extracted from leaf tissue using the CTAB method (Doyle and Doyle 1987). The voucher specimen (Voucher number: 08CS573, Collected By Ting Zhang, zhangting@mail.kib.ac.cn) was deposited in the Herbarium of the Kunming Institute of Botany, Chinese Academy of Sciences, Yunnan, China (KUN). Genome skimming sequencing was performed on the Illumina HiSeq X Ten platform (Illumina, San Diego, CA). Totally, 3.15 Gb high-quality clean data were generated. The GetOrganelle v1.7.0 (Jin et al. 2020) pipeline was used to assemble the complete plastid genome with clean sequencing data. The Bandage v 0.8.1 (Wick et al. 2015) was used to visualize the completeness of genome assembly. Finally, the PGA (Qu et al. 2019) was used to annotate all genes of the plastid genome with the whole plastid genome sequence of *Scutellaria lateriflora* (NC_034693) as reference. The newly annotated plastid genome was available in GenBank of NCBI (accession number: MZ954872.1).

The complete plastid genome of *S. microviolacea* was 152,092 bp in length and comprised of a pair of inverted repeats (IRs) of 25,234 bp each, a large single-copy (LSC) region of 84,090 bp, and a small single-copy (SSC) region of 17,534 bp. The overall GC content of this genome was 38.3% (IRs, 43.6%; LSC, 36.4%; SSC, 32.5%). Totally, the plastid genome encoded 131 genes, including 87 protein-coding genes, 36 tRNA genes, and 8 rRNA genes.

To clarify the phylogenetic position of *S. microviolacea*, 16 plastomes involving 14 species from the genus of *Scutellaria* were used to perform the maximum likelihood (ML) analysis (Figure 1). Due to the successive sister groups’ relationship among *Tinnea*, *Holmskioldia*, and *Scutellaria* (Zhao et al. 2021), we chose *Tinnea aethiopica*, *Holmskioldia sanguinea* as outgroups. All plastid genomes were aligned by MAFFT (Katoh and Standley 2013) plugin in the software PhyloSuite (Zhang et al. 2020). The ML analysis was performed using RAxML (Stamatakis 2006) under the GTR + G model, and the rapid bootstrap was set to 1000 replicates. Based on our phylogeny, the genus *Scutellaria* was divided into two clades, which is consistent with previous studies despite different samplings being used (Safikhani et al. 2018; Zhao et al. 2021). The endemic species *S. microviolacea* is evolutionarilyclose to *Scutellaria tsinyunensis*. Given the species richness and complex taxonomy history in the genus *Scutellaria*, sufficient species sampling with genomic sequencing data is necessary to accurately authenticate the interspecific relationship of *Scutellaria*. The newly characterized complete plastid genome of *S. microviolacea* will provide essential resources for further studies on the evolution and genetic diversity of the genus *Scutellaria* and its related taxa.

**Figure 1.**
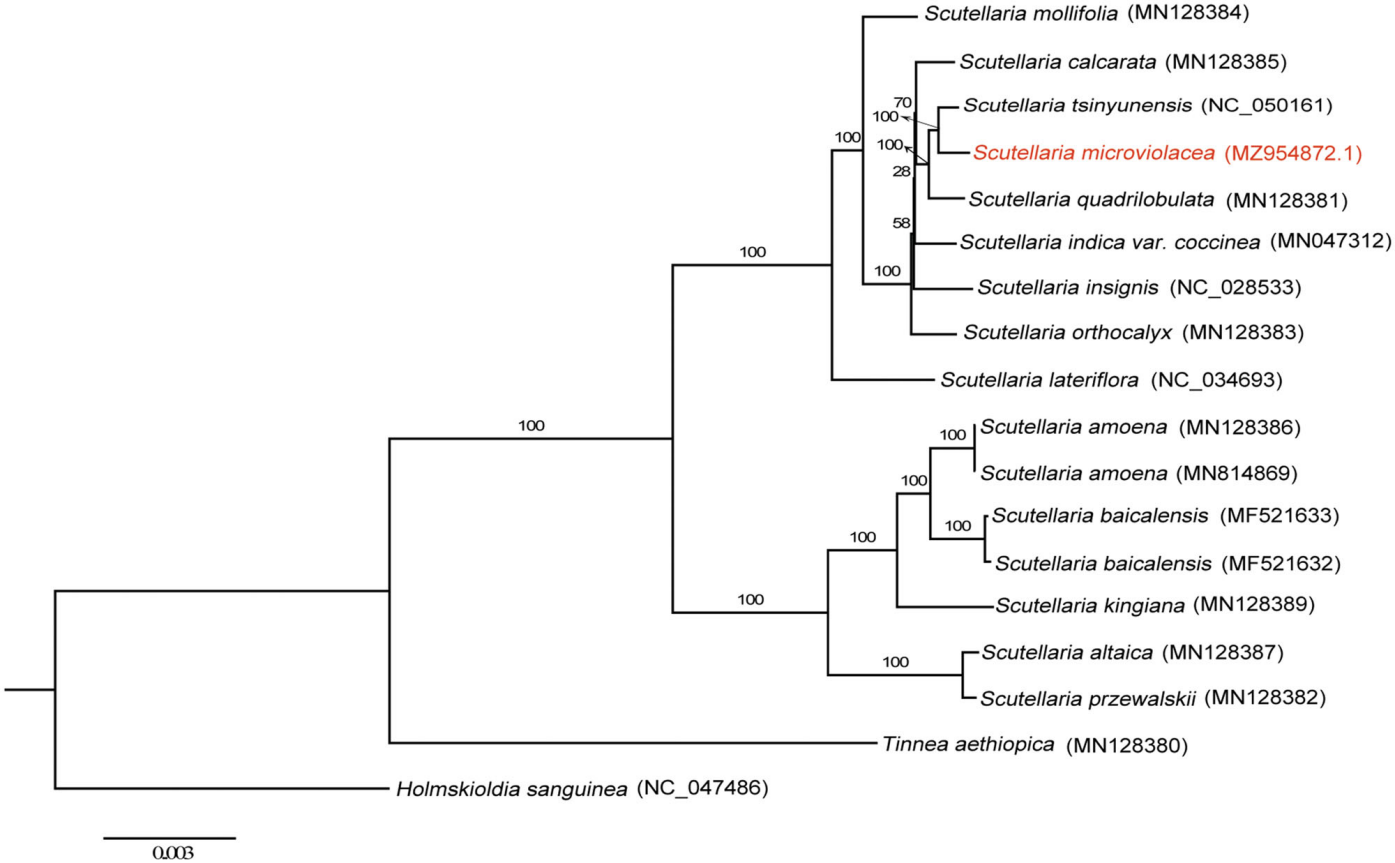
The maximum-likelihood (ML) tree was based on complete plastid genome sequences from 14 species of *Scutellaria*. *Tinnea aethiopica* (MN128380) and *Holmskioldia sanguinea* (NC_047486) were used as outgroups. The bootstrap support values with 1000 replicates were shown at each node.

## Ethical approval

The sample collection was authorized by Professor Jun-Bo Yang (Germplasm Bank of Wild Species, Kunming Institute of Botany, CAS).

## Author contributions

YC Wang drafted the manuscript; YC Wang and ZR Zhang performed the data analysis; LJ Yan designed this study and revised the manuscript critically for intellectual content; all authors contributed to the final approval of the version to be published and all agree to be accountable for all aspects of the work.

## Data Availability

The genome sequence data that support the findings of this study are openly available in GenBank of NCBI at (https://www.ncbi.nlm.nih.gov/) under the accession no. MZ954872.1. The associated BioProject, Bio-Sample and SRA, numbers are PRJNA758007, SAMN20989313, and SRR15672851 respectively.
